# Federated Multi-View Unsupervised Feature Selection via Bio-Inspired Hierarchical-Cognitive Tianji’s Horse Racing Optimization and Tensor Learning

**DOI:** 10.3390/biomimetics11050312

**Published:** 2026-05-01

**Authors:** Rong Cheng, Zhiwei Sun, Kun Qi, Wangyu Wu, Lingling Xu

**Affiliations:** 1 School of Electronic and Communication Engineering, Shenzhen Polytechnic University, Shenzhen 518055, China; chengr@szpu.edu.cn (R.C.); qikun@szpu.edu.cn (K.Q.); 2 Undergraduate School of Artificial Intelligence, Shenzhen Polytechnic University, Shenzhen 518055, China; smeker@szpu.edu.cn; 3 School of Computer Science, University of Liverpool, Liverpool L69 3DR, UK; wangyu.wu@liverpool.ac.uk; 4 School of Computer Science and Engineering, South China University of Technology, Guangzhou 510006, China

**Keywords:** federated learning, multi-view unsupervised feature selection, Tianji’s horse racing optimization, bio-inspired computation, tensor learning, privacy preservation

## Abstract

As multi-view datasets expand across diverse practical fields, feature selection (FS) has become an indispensable preparatory stage for machine learning models. Nevertheless, real-world multi-view data is often unlabeled and distributed among isolated clients, posing significant challenges to traditional centralized methods due to privacy concerns and communication constraints. Furthermore, existing centralized and federated approaches frequently suffer from entrapment in local optima and lack robust convergence guarantees. To address these issues, we propose Fed-MUFSHT, a federated framework for multi-view unsupervised FS (MUFS) that integrates tensor learning with a novel metaheuristic optimizer, Hierarchical-Cognitive Tianji’s Horse Racing Optimization (HC-THRO). Within the federated learning paradigm, Fed-MUFSHT follows a dual-stage local optimization process. Stage 1 applies HC-THRO, which integrates Hierarchical Competitive Learning and Adaptive Cognitive Mapping to simulate multi-level strategic competition and cognitive adaptation among individuals. This design enhances global exploration, adaptive learning, and fine-grained feature selection in high-dimensional spaces. Stage 2 employs a TL module based on canonical polyadic (CP) decomposition to perform missing-view imputation and refine latent representation learning. At the global level, a privacy-preserving aggregation strategy based on Normalized Mutual Information (NMI) and feature weights enables efficient model coordination without exposing raw data. Comparative experiments on several public benchmark datasets reveal that Fed-MUFSHT maintains clear advantages over strong competing methods, showing better optimization results together with more dependable convergence characteristics. The overall evidence suggests that the proposed approach is both robust and effective for distributed optimization tasks involving privacy protection.

## 1. Introduction

Multi-view information is increasingly prevalent across diverse practical domains, where individual instances are represented via non-uniform features gathered from distinct angles. Such cross-view features frequently demonstrate interdependent and mutually beneficial characteristics [1,2]. Moreover, owing to the heterogeneity of feature sources and the high cost associated with data annotation, integrated datasets commonly exhibit high-dimensional structures with insufficient labeling [3]. In the era of big data, the task of discovering informative features from unlabeled multi-view data is of great practical significance for areas such as social network analysis [4], image recognition [5], recommendation services, RNA–small molecule interaction modeling [6], and industrial bearing life prediction [7]. Multi-view Unsupervised Feature Selection (MUFS) has risen to prominence as a mature strategy for extracting discriminative attributes from unlabeled, multi-perspective data. Its goal is to reduce dimensionality and remove redundant features, thereby improving the performance of downstream tasks [8,9].

Notwithstanding the effectiveness of these methods, a fundamental limitation lies in their reliance on the assumption of centralized data availability. In practical scenarios, such as cross-institutional healthcare and inter-bank finance, multi-view data is inherently dispersed across isolated clients [10]. Driven by escalating worries over data confidentiality and strict legal mandates—most notably the GDPR—the direct sharing of original datasets across decentralized nodes has become heavily restricted, resulting in fragmented and isolated data repositories [11]. As a result, conventional centralized approaches for MUFS are no longer viable within these distributed settings. In this context, federated learning (FL) offers a promising alternative that ensures privacy by enabling joint model optimization while keeping local data stored onsite, thus eliminating the need to transmit raw information [12]. This secure collaborative paradigm is further supported by recent value-iteration approaches in multi-agent systems [13]. Nevertheless, extending the FL framework to address Multi-view Unsupervised Feature Selection tasks introduces substantial technical challenges. Specifically, data distributed across clients often displays statistical heterogeneity (i.e., Non-IID distributions) and is frequently plagued by incomplete view coverage [14]. Moreover, in the absence of label supervision, federated optimization methods are highly prone to instability and premature convergence to suboptimal solutions.

Current approaches for Multi-view Unsupervised Feature Selection (MUFS), mostly designed for central processing, fall naturally into two distinct categories. The first category transforms heterogeneous multi-perspective inputs into a unified format—often via straightforward fusion like vector stacking—before applying standard FS algorithms developed for single-view data. Representative methods include SPCAFS [15], GBUFS [16], and HSL [17]. While computationally lightweight and easy to implement, these strategies often fail to adequately model the hidden interdependencies among different views. The second category performs feature selection while explicitly preserving the multi-view structure, with a focus on mining the underlying relationships across views. Typical examples include JMVFG [18], CDMvFS [19], and SDFS [20]. These techniques typically employ graph-based modeling—such as constructing affinity matrices or leveraging graph neural networks—to enforce alignment among multiple views, which in turn boosts the quality of selected features. However, since their exploitation of cross-view relational information is still limited, the quality of the selected features remains suboptimal. More importantly, these centralized approaches cannot address the privacy constraints and communication barriers inherent in distributed environments.

In distributed multi-view settings, unsupervised feature selection faces even more practical challenges. Take medical diagnosis as an example: a patient is often associated with multiple views such as clinical text records, imaging examinations, and various laboratory indicators. In a realistic distributed scenario, these views might be stored separately in different hospital departments or partner institutions. While semantically related, they exhibit markedly different statistical properties. In the absence of labels, it remains difficult to automatically identify truly discriminative features from such heterogeneous, distributed sources while preserving privacy. Although existing methods such as MIMB [21] and IMC-MCL [22] can exploit multi-view structural information to some extent, they typically treat data recovery, feature selection, and representation learning as separate stages, failing to exploit their potential synergy within a unified framework. By employing a joint optimization strategy, TIME-FS [23] seeks to combine FS with the acquisition of low-dimensional representations. However, in terms of fine-grained modeling of multi-view correlations, the discriminativeness of selected features, and the optimization efficiency on high-dimensional data—especially under the constraints of federated communication—there is still considerable room for improvement.

To address the optimization instability and premature convergence often encountered in high-dimensional unsupervised tasks, researchers have increasingly turned to bio-inspired and meta-heuristic algorithms [24]. To strengthen global searching efficiency, researchers have utilized various techniques, including the Superb Fairy-wren Optimization (SFOA) [25], the iHow Optimization method (IOA) [26], and Particle Swarm Optimization (PSO) [27] contributes to a more effective exploration of the overall search space. Drawing inspiration from the classical Chinese tactical concept of “Tianji’s horseracing,” Wang et al. [28] developed a novel meta-heuristic entitled Tianji’s Horse Racing Optimization (THRO), which focuses on adaptive competition and resource allocation to win under disadvantageous conditions. THRO models the dynamic competition between two opposing populations, simulating multi-level strategic confrontations and cooperative co-evolution to equilibrate the relationship between broad exploration and intensified exploitation. Specifically, this approach introduces a competitive game mechanism and a greedy matching strategy, enabling individuals to adaptively engage in asymmetric contests that enhance search diversity and convergence accuracy. Incorporating the core mechanisms of THRO into MUFS is expected to further strengthen the MUFS model’s global search ability and convergence stability. Embedding this powerful optimization strategy within a federated framework is crucial for overcoming local-optimum stagnation commonly encountered in distributed unsupervised learning.

In response to these challenges, we propose Fed-MUFSHT, a privacy-preserving federated framework for multi-view unsupervised feature selection (MUFS). Fed-MUFSHT features a local–global collaborative design. On each client, a two-stage local optimization is performed: Stage-1 applies Hierarchical-Cognitive THRO (HC-THRO) to enhance global exploration and avoid local optima in high-dimensional feature spaces; Stage-2 employs tensor learning (TL) based on canonical polyadic (CP) decomposition to recover latent representations and impute missing views. At the server, an NMI-guided adaptive weighting mechanism securely fuses client updates to support joint optimization without exchanging raw data, thereby reconciling privacy requirements with effective FS.

The key contributions of this paper are listed as follows:

- We present Fed-MUFSHT, a privacy-preserving federated MUFS framework that enables collaborative feature selection over distributed multi-view data under the “data silo” constraint.
- We develop a two-stage client-side optimization scheme: (i) HC-THRO integrates hierarchical competition with cognitive adaptation to improve exploration–exploitation balance and achieve fine-grained selection in high-dimensional spaces; (ii) a CP-based TL module performs missing-view imputation and latent representation refinement to better exploit cross-view dependencies.
- We design an NMI-guided secure aggregation strategy with adaptive feature weighting, which stabilizes federated optimization and yields superior solution quality compared with state-of-the-art centralized and federated baselines.

The rest of this paper is organized as follows. Section 2 reviews representative single-view and multi-view unsupervised FS methods, highlighting their core ideas, methodological advances, and remaining limitations. Section 3 presents the proposed framework, including its theoretical foundations and formal problem formulation. Section 4 reports extensive experimental results and comparative evaluations. Finally, Section 5 concludes the paper and discusses directions for future work.

## 2. Related Work

A comprehensive survey of the pivotal literature concerning single-view and multi-view unsupervised FS is presented in this section, summarizing their core mechanisms, methodological innovations, and prevailing limitations.

### 2.1. Single-View Unsupervised FS

Unsupervised FS under a single-view setting is commonly implemented through one of three core frameworks: filters that score features independently, wrappers that evaluate subsets via model performance, or embedded methods that integrate selection within the learning process.

Filter-based approaches prioritize the evaluation of feature distinctiveness via predefined statistical metrics, such as variance, to eliminate redundancy. Representative instances include the Laplacian Score (LS) [29], which gauges merit based on the preservation of local manifold structures, and Spectral FS (SPEC) [30], which employs graph spectral theory to rank feature significance by fusing supervised and unsupervised criteria. Although computationally efficient, these methods are often constrained by their focus on intrinsic sample attributes, frequently neglecting latent inter-feature dependencies.

Conversely, wrapper methodologies [31] treat subset search as an iterative process, assessing subset quality by training specific learning algorithms. While typically yielding higher accuracy than filters, their practical utility in large-scale scenarios is curtailed by prohibitive computational overheads.

In contrast, embedded strategies integrate FS directly into the optimization objectives of model training, facilitating synergistic improvement. To illustrate, Wang et al. [32] developed an architecture that integrates local structural analysis with a sparse regression model using exponential weighting, mitigating the tendency of conventional techniques to over-prioritize high-weight attributes while simultaneously preserving both large-scale and regional data characteristics. The FBUFS algorithm was proposed by Li et al. [33], utilizing binary differential evolution alongside pseudo-labels and local manifold structures to optimize feature weight learning. Fan et al. [34] put forward a beluga whale optimizer incorporating multiple strategic improvements (MSBWO), which boosted both the quality of selected feature subsets and the speed at which the algorithm converged. To tackle the difficulties posed by high-dimensional classification tasks, Miao et al. [35] designed a variant of WOA that integrates dynamic multi-strategy mechanisms alongside elite-guided tuning. Meanwhile, Pramanik [36] demonstrated that a WOA-driven deep feature selection framework (U-WOA) performs effectively in identifying cancerous regions within breast ultrasound scans. Furthermore, the Structured Optimal Graph (SOGFS) was designed by Nie et al. [37] to capture local structures via adaptive sample similarity learning while synchronously performing feature screening.

Nevertheless, when these single-view techniques are directly transposed to multi-view data, simple feature concatenation is typically employed. This practice severs intrinsic correlations between views, making it difficult to mine complementary information, thereby diminishing the overall efficacy of feature selection.

### 2.2. Multi-View Unsupervised FS

Unlike paradigms relying on a single data view, MUFS methods aim to uncover latent inter-view correlations and complementary characteristics, ultimately yielding more robust and representative feature subsets.

To improve human action retrieval, Wang et al. [38] introduced the adaptive AMFS method, which exploits complementary information across multiple views by constructing view-specific Laplacian matrices with dynamically assigned weights. Cao et al. [39] innovatively incorporated high-order neighbor information into the graph construction process, mapping data to a shared latent space to capture feature-level complementarity. Subsequently, Cao et al. [19] designed a strategy for generating mutually exclusive multi-view graphs, combining graph learning with consistent clustering to alleviate interference caused by heterogeneous views. Liu et al. [40] proposed a coherent framework based on hidden semantic representations and anchor-driven graph modeling, where feature weighting and redundancy control are jointly considered to lower the computational burden of large graph structures. To address data imbalance and incompleteness, Yang et al. [41] introduced TERUIMUFS, which fuses self-representation learning with tensor low-rank constraints to correct structural errors. Similarly, Yu et al. [42] reconstructed missing information in the latent space through dual-layer local similarity graphs while simultaneously improving feature selection and data reconstruction using a linear-complexity optimization strategy. Huang et al. [23] proposed TIME-FS, a tensor-decomposition-based framework that jointly handles missing-value imputation, discriminative feature selection, and low-dimensional representation learning, thereby markedly decreasing computational cost. Recently, Yuan et al. [43] made an early attempt to extend Fed-IMUFS to the MUFS setting. Although this method combines a fractional sparsity-guided whale optimization algorithm with tensor alternating learning, deficiencies remain regarding the quality of the FS solution and convergence stability.

Despite these significant advancements, two major bottlenecks persist: (1) existing methods predominantly rely on centralized assumptions, complicating their adaptation to privacy-sensitive distributed scenarios characterized by “data silos”; and (2) optimization algorithms still leave room for improvement in solution quality and convergence performance. Addressing these challenges, the improved THRO (HC-THRO) is introduced in this study, building upon TIME-FS to enhance global search and convergence capabilities. Furthermore, the method is implemented under a federated learning framework and leverages a secure aggregation mechanism based on NMI and feature weighting, thereby addressing privacy protection and feature-selection challenges in distributed scenarios. To further clarify the positioning and novelty of our approach relative to the most relevant prior works, Table 1 presents a structured comparison of Fed-MUFSHT against the two most closely related methods: TIME-FS [23] (the state-of-the-art centralized MUFS method) and Fed-IMUFS [43] (the only existing federated MUFS method).

As shown in Table 1, the novelty of Fed-MUFSHT is not merely a combination of existing components, but rather a fundamentally new synergistic architecture that addresses critical limitations of prior works. Specifically, (1) compared with TIME-FS, Fed-MUFSHT introduces HC-THRO as a powerful global search engine to overcome the local-optima entrapment inherent in pure alternating optimization and extends the entire pipeline to a privacy-preserving federated setting; (2) compared with Fed-IMUFS, Fed-MUFSHT replaces the sparsity-guided WOA with the novel HC-THRO, which features dedicated HCL and ACM mechanisms for a more principled balance between exploration and exploitation, and further introduces a secure aggregation strategy utilizing NMI and adaptive feature weighting that surpasses simple weighted averaging. Most critically, the tight, closed-loop coupling between the outer-layer HC-THRO and the inner-layer TL—where the metaheuristic’s global solution directly initializes the tensor learning module, and the tensor learning module’s refined output feeds back to update the evolutionary population—creates a unique optimization dynamic that is absent in both TIME-FS and Fed-IMUFS.

## 3. The Proposed Method

In this section, we provide the implementation details of the proposed Fed-MUFSHT. Specifically, under the FL setting, each client performs an outer-layer optimization procedure based on HC-THRO and an inner-layer optimization mechanism driven by TL, while the server carries out a secure multi-party aggregation and distribution strategy guided based on NMI and feature weights.

### 3.1. Client-Side Operations

#### 3.1.1. MUFS Matrix Initialization

In the client-side stage, the dataset is represented by multiple views

(1) $$X=\{X^{\left(1\right)},\ldots ,X^{\left(V\right)}\}.$$

Here, $X^{\left(v\right)}\in R^{d_{v}\times n}$ is the data matrix of the *v*-th view, containing *n* observations and $d_{v}$ attributes. For each view, a matrix $W^{\left(v\right)}\in R^{d_{v}\times c}$ is introduced to select informative variables and transform them into a common *c*-dimensional subspace. Typically, *c* is determined by the number of clusters or semantic categories. Therefore, the total number of variables to be optimized over all views is given by

(2) $$\dim\left(W\right)=\sum_{v=1}^{V}d_{v}c.$$

For initialization, each $W^{\left(v\right)}$ is obtained by reshaping a randomly sampled vector $w_{\mathrm{rec}}^{\left(v\right)}\in R^{d_{v}c}$ into a matrix:

(3) $$W^{\left(v\right)}=\mathrm{Fold}\;\left(w_{\mathrm{rec}}^{\left(v\right)},\;d_{v},\;c\right),$$

where $\mathrm{Fold}(\cdot ;\;d_{v},\;c)$ converts a vector of length $d_{v}c$ into a $d_{v}\times c$ matrix in row-major order. The overall feature selection representation for the multi-view data is $W=\{W^{\left(1\right)},\ldots ,W^{\left(V\right)}\}$.

#### 3.1.2. Outer-Layer Optimization Based on HC-THRO

In the outer layer, we employ HC-THRO, a population-based metaheuristic that simulates multi-level competitive evolution between two interacting populations. This algorithm integrates the concepts of hierarchical competition and cognitive adaptation to strike an effective trade-off between global search capabilities and local refinement precision.

For the *i*-th individual of population *A* (representing Tianji’s horses) at iteration *t*, its position is updated through a competitive interaction with one opponent in population *B* (the King’s horses) according to the Hierarchical Competitive Learning (HCL) mechanism:

(4) $$x_{i}^{t+1}=x_{i}^{t}+\alpha_{t}\;R_{1}\;\left(x_{\mathrm{best},B}^{t}-x_{i}^{t}\right)+\beta_{t}\;R_{2}\;\left(x_{\mathrm{rand},A}^{t}-x_{i}^{t}\right),$$

where $\alpha_{t}$ and $\beta_{t}$ are adaptive learning coefficients controlling inter-population competition and intra-population exploration, respectively. $R_{1},R_{2}$∼Uniform(0,1) are independent random matrices; $x_{\mathrm{best},B}^{t}$ denotes the fittest opponent in population *B*; and $x_{\mathrm{rand},A}^{t}$ is a randomly selected peer from the same population. This formulation embodies the “race” dynamics where weaker individuals learn from stronger ones, thereby enhancing hierarchical self-learning and maintaining population diversity.

To further refine individual behavior, the Adaptive Cognitive Mapping (ACM) strategy introduces historical memory and population cognition into the search. Each individual updates its position as:

(5) $$x_{i}^{t+1,\mathrm{ACM}}=x_{i}^{t+1}+\gamma_{t}\;\left({\mathrm{pbest}}_{i}-x_{i}^{t}\right)+\delta_{t}\;\left({\mathrm{gmean}}^{t}-x_{i}^{t}\right),$$

where ${\mathrm{pbest}}_{i}$ is the historical best position of individual *i*; ${\mathrm{gmean}}^{t}=\frac{1}{N}\sum_{j=1}^{N}x_{j}^{t}$ represents the cognitive centroid of population *A*; and $\gamma_{t}$, $\delta_{t}$ are cognitive adaptation factors controlling the levels of memory reinforcement and cooperative adjustment. Through ACM, individuals balance exploitation around personal experience with cooperation guided by collective knowledge, thus avoiding premature convergence and improving search stability.

The synergy between HCL-based strategic competition and ACM-based cognitive refinement allows HC-THRO for maintaining a flexible equilibrium between wide-ranging search and precise local tuning, allowing agents to break free from suboptimal traps.

After each iteration, the global best individual $x_{\mathrm{gbest}}$ is regarded as the current optimal FS solution, which is then reshaped into view-specific matrices $W^{\left(v\right)}$ following the initialization scheme described in Section 2 (using the same folding operation as in Equation (3)).

#### 3.1.3. Inner-Layer Optimization Mechanism Based on Tensor Learning

After each outer-layer optimization, the globally searched $W^{\left(v\right)}$ is used as prior information to guide the update of the remaining variables in the inner layer. The inner refinement module, inspired by TIME-FS [23], employs a structured tensor decomposition framework to perform missing-view reconstruction, representation learning, and view alignment, with adaptive modifications made to the original model. Once the inner-layer variables are updated, their outputs are fed back to the outer layer to further adjust and drive the subsequent evolutionary feature selection process.

(1) Missing-View Completion. Drawing from the imputation scheme in TIME-FS [23], we incorporate a strategy for filling gaps across views. Denote the partial data matrix by $X^{o}\in R^{d\times n}$, where *d* represents the feature count and *n* the number of instances. To precisely indicate absent values, an indicator matrix $G^{o}\in {\left\{0,1\right\}}^{d\times n}$ is employed, such that $G_{ij}^{o}=1$ if the entry $X_{ij}^{o}$ is available, and 0 otherwise. This mask can be formulated as

(6) $${\hat{X}}^{\left(v\right)}=E^{\left(v\right)}⊙G^{\left(v\right)}+X^{\left(v\right)},$$

where $E^{\left(v\right)}$ is a learnable residual (imputation) matrix and ⊙ denotes the (element-wise) product.

(2) Joint Embedding and View Weighting. For each view *v*, we learn a low-dimensional representation $Z^{\left(v\right)}\in R^{k\times n}$ together with a feature selector $W^{\left(v\right)}\in R^{d_{v}\times k}$. The joint objective is formulated as

(7) $$\min_{\left\{W^{\left(v\right)},\;Z^{\left(v\right)},\;\alpha_{v}\right\}}\sum_{v=1}^{V}\beta \sum_{v=1}^{V}{∥W^{\left(v\right)}∥}_{2,1}+\lambda \;\mathrm{tr}\;\left(\left({\left(W^{\left(v\right)}\right)}^{⊤}QW^{\left(v\right)}\right)\right)+\alpha_{v}{∥{\hat{X}}^{\left(v\right)}-W^{\left(v\right)}Z^{\left(v\right)}∥}_{F}^{2},$$

where ${∥\cdot ∥}_{F}$ is the Frobenius norm, ${∥\cdot ∥}_{2,1}$ enforces row-wise sparsity, Q encodes structural priors (e.g., a graph Laplacian), and λ,β>0 are regularization coefficients. The view weight $\alpha_{v}$ is adaptively determined from the reconstruction error and normalized such that views with smaller errors receive larger weights; a temperature parameter γ∈(0,1) is used to control the sharpness of the weight distribution.

(3) Tensor CP Decomposition. To capture cross-view dependencies, representations from each view are assembled into a three-way tensor $Z=\left[Z^{\left(1\right)},\ldots ,Z^{\left(V\right)}\right]\in R^{k\times n\times V}$, where concatenation is along the view mode. The tensor is then approximated via a rank-*r* CP factorization:

(8) $$Z\approx ⟦M,P,A⟧=\sum_{j=1}^{r}m_{j}\circ p_{j}\circ a_{j}.$$

Here, $M\in R^{V\times r}$ encodes view-level coefficients, $P\in R^{n\times r}$ describes sample–anchor affinities, $A\in R^{k\times r}$ represents anchor bases in the embedding space, ∘ denotes the outer product, and ⟦·⟧ denotes the CP factors.

(4) Anchor Graph Learning. The CP factors are updated via alternating least squares (ALS) over different tensor modes. Let $Z_{\left(m\right)}$ be the mode-*m* unfolding of Z. The updates are

(9) $$\begin{array}{cc} \;\;\;\;\;P & =Z_{\left(2\right)}\left(A⊙M\right){\left({\left(A⊙M\right)}^{⊤}\left(A⊙M\right)\right)}^{-1}, \end{array}$$

(10) $$\begin{array}{cc} \;\;\;\;\;A & =Z_{\left(1\right)}\left(M⊙P\right){\left({\left(M⊙P\right)}^{⊤}\left(M⊙P\right)\right)}^{-1}, \end{array}$$

(11) $$\begin{array}{cc} \;\;\;\;\;\;\;Y & =-Z_{\left(3\right)}\left(A⊙P\right){\left(\eta I+{\left(A⊙P\right)}^{⊤}\left(A⊙P\right)\right)}^{-1}, \end{array}$$

(12) $$\begin{array}{cc} M & =\max\;\left(\frac{1+\gamma Y}{r}1^{⊤}-Y,\;0\right), \end{array}$$

where η>0 controls the sparsity of M, I is the identity matrix, and 1 is an all-ones vector used to enforce non-negativity and row normalization.

(5) Missing-value Refinement. After representation learning, the missing entries are further adjusted by

(13) $$E^{\left(v\right)}=G^{\left(v\right)}⊙\left({\hat{X}}^{\left(v\right)}-Z^{\left(v\right)}W^{\left(v\right)}\right).$$

(6) Feature Selector Synchronization. To keep the inner model aligned with the outer optimizer, the internal feature selectors $W^{\left(v\right)}$ are periodically updated toward the best view-specific solution $W_{\mathrm{best}}^{\left(v\right)}$ found by HC-THRO, ensuring that each view’s selector remains synchronized with the current global search state.

(7) Overall Objective Function. Finally, the inner module optimizes a unified objective combining reconstruction loss, structural and sparsity regularization, imputation consistency, and CP-based alignment:

(14) $$\begin{array}{cc} \min_{\{W^{\left(v\right)},E^{\left(v\right)},Z^{\left(v\right)}\},\;M,P,A,\;\alpha }\; & \sum_{v=1}^{V}\mu \sum_{v=1}^{V}{∥E^{\left(v\right)}∥}_{F}^{2}+\lambda \sum_{v=1}^{V}\mathrm{tr}\;\left({\left(W^{\left(v\right)}\right)}^{⊤}QW^{\left(v\right)}\right)\\ \; & \;+\beta \sum_{v=1}^{V}{∥W^{\left(v\right)}∥}_{2,1}+\alpha_{v}{∥{\hat{X}}^{\left(v\right)}-Z^{\left(v\right)}W^{\left(v\right)}∥}_{F}^{2}+\tau {∥Z-⟦M,P,A⟧∥}_{F}^{2},\\ s.t.\; & \alpha_{v}\geq 0,\;\sum_{v=1}^{V}\alpha_{v}=1,\;M1=1,\;M\geq 0. \end{array}$$

Here, ${\hat{X}}^{\left(v\right)}$ denotes the completed data of view *v*; $W^{\left(v\right)}$ selects and reweights features; and $Z^{\left(v\right)}$ provides the low-dimensional representation. The coefficients $\alpha_{v}$ form an adaptive view-weight vector with $\sum_{v}\alpha_{v}=1$. Matrix Q encodes prior structural information, and λ,β,μ,τ>0 control, respectively, structure preservation, sparsity, imputation penalty, and CP reconstruction fidelity. The CP factors are denoted as (A,P,M), where $M\in R^{V\times r}$ is required to be non-negative and row-normalized, i.e., M1=1.

(8) Update Strategy. All variables (i.e., $Z^{\left(v\right)}$, A, P, M, $E^{\left(v\right)}$, and $W^{\left(v\right)}$) are updated in an alternating manner until convergence. This coordinated inner loop supports robust missing-view completion, discriminative embedding, and view-aligned representation, thereby improving the overall performance of MUFS.

**Figure 1:**
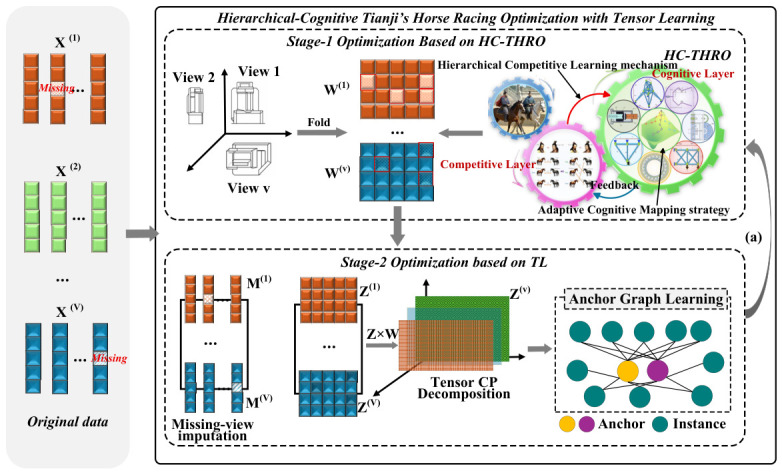
Theworkflow of the proposed MUFSPCTL. (a) Convergence of the objective function is evaluated by computing it according to Equation (14).

The specific implementation of MUFSPCTL is shown in Figure 1.

### 3.2. Server-Side Operations

#### 3.2.1. A Secure Aggregation Strategy Utilizing NMI and Adaptive Feature Weighting

**Figure 2:**
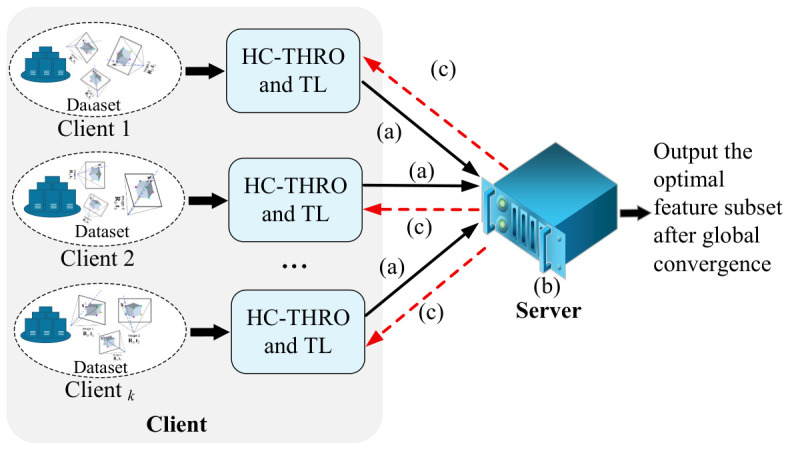
The framework of Fed-MUFSHT includes a secure multi-party aggregation and distribution strategy based on NMI and feature weights, implemented as follows: (a) client *k* performs local HC-THRO/TL on private data to obtain $\left(W_{k}^{\left(r\right)},q_{k}^{\left(r\right)}\right)$; (b) only privacy-preserving uploads (e.g., encrypted/secret-shared) of $\left\{W_{k}^{\left(r\right)},q_{k}^{\left(r\right)}\right\}$ are sent to the server (raw data never leaves clients); (c) the server computes NMI-based weights $\gamma_{k}^{\left(r\right)}$ and conducts secure aggregation ${\tilde{W}}^{\left(r\right)}=\sum_{k\in S^{\left(r\right)}}\gamma_{k}^{\left(r\right)}W_{k}^{\left(r\right)}$, then broadcasts ${\tilde{W}}^{\left(r\right)}$.

Within the federated framework of Fed-MUFSPT (as shown in Figure 2), data security and privacy preservation are of primary concern. To this end, we design a secure aggregation strategy utilizing NMI and adaptive feature weighting. Specifically, the server aggregates and redistributes only (i) the NMI scores computed from the selected features and (ii) the corresponding optimal feature-weight matrices uploaded by each client, without accessing or collecting any raw data. Meanwhile, the original datasets remain strictly on local clients.

Step 1: Client upload (inputs to the server). At the end of communication round *r*, each participating client $k\in S^{\left(r\right)}$ uploads the pair $\left\{W_{k}^{★(r)},q_{k}^{\left(r\right)}\right\}$, where $W_{k}^{★(r)}\in R^{D\times c}$ denotes the locally optimized feature-weight matrix (concatenated across views if necessary), and $q_{k}^{\left(r\right)}$ is the clustering-quality score measured by NMI:

(15) $$q_{k}^{\left(r\right)}=\mathrm{NMI}\;\left(y_{k}^{\left(r\right)},\;{\hat{y}}_{k}^{\left(r\right)}\right),$$

where $y_{k}$ and ${\hat{y}}_{k}$ denote two cluster assignments (e.g., predicted labels versus reference labels or pseudo-labels) obtained by the local unsupervised pipeline using the selected features. The specific construction of $y_{k}$ and ${\hat{y}}_{k}$ follows the same evaluation protocol used in the experiments.

Step 2: NMI-based secure weighting. Once all client submissions are gathered, the server derives fusion coefficients tailored to each participant. To improve robustness and suppress low-quality updates, a soft weighting rule with temperature τ>0 is adopted:

(16) $$\gamma_{k}^{\left(r\right)}=\frac{\exp\;\left(q_{k}^{\left(r\right)}/\tau \right)}{\sum_{j\in S^{\left(r\right)}}\exp\;\left(q_{j}^{\left(r\right)}/\tau \right)},\;\sum_{k\in S^{\left(r\right)}}\gamma_{k}^{\left(r\right)}=1.$$

When τ→∞, Equation (16) reduces to uniform averaging, whereas when $\tau \to 0^{+}$, the aggregation increasingly concentrates on the best-performing clients. Alternatively, a linear normalization can be adopted: $\gamma_{k}=q_{k}/\sum_{j\in S}q_{j}$.

Step 3: Secure aggregation of feature-weight matrices. The global feature-weight matrix is obtained via weighted aggregation:

(17) $${\tilde{W}}^{\left(r\right)}=\sum_{k\in S^{\left(r\right)}}\gamma_{k}^{\left(r\right)}\;W_{k}^{★(r)}.$$

Since only model parameters $W_{k}^{★(r)}$ and scalar scores $q_{k}^{\left(r\right)}$ are used, raw data never leaves the clients, which preserves privacy by design.

Step 4: Feature-weight-based distribution (personalized initialization). To further exploit the aggregated model while respecting client heterogeneity, the server distributes a personalized initialization to each client:

(18) $$W_{k}^{0(r+1)}=\eta \;{\tilde{W}}^{\left(r\right)}+\left(1-\eta \right)\;W_{k}^{★(r)},\;0\leq \eta \leq 1,$$

where η controls the degree of global guidance. The matrix $W_{k}^{0(r+1)}$ is then used to initialize the next-round client-side optimization (e.g., as the folded representation of the best particle in HC-THRO or as the initial feature selector in the tensor learning module).

Step 5: Early stopping criterion. An early stopping rule can be triggered if the improvement of the global quality indicator becomes sufficiently small:

(19) $$\left|{\bar{q}}^{\left(r\right)}-{\bar{q}}^{\left(r-1\right)}\right|<\epsilon ,\;{\bar{q}}^{\left(r\right)}=\frac{1}{|S^{\left(r\right)}|}\sum_{k\in S^{\left(r\right)}}q_{k}^{\left(r\right)},$$

where ϵ is a predefined threshold.

#### 3.2.2. Privacy Analysis

Threat model. Our framework operates under the standard honest-but-curious (or semi-honest) threat model, where the central server and clients follow the protocol correctly but may attempt to infer private information from the messages they receive. Each client’s raw data $\left\{X_{k}^{\left(v\right)}\right\}$ remains strictly on-device. The only information transmitted to the server is the locally optimized feature-weight matrix $W_{k}^{★(r)}$ and a scalar quality score $q_{k}^{\left(r\right)}$.

Privacy Leakage Risk Assessment. From the server’s perspective, reconstructing raw data from the uploaded $W_{k}^{★(r)}\in R^{D\times c}$ is computationally infeasible. This is because it requires solving a severely under-determined inverse problem, as the sample dimension *n* (where typically c≪n) is completely absent from the uploaded parameters. Furthermore, to prevent the server from inspecting even the individual updates, the aggregation in Equation (17) can be implemented using standard secure aggregation protocols (e.g., additive secret sharing [44]), which would allow the server to only see the final aggregated model ${\tilde{W}}^{\left(r\right)}$.

Compatibility with differential privacy. To provide formal privacy guarantees, Fed-MUFSHT is fully compatible with the (ε,δ)-differential privacy (DP) framework [45]. Although not enabled by default in our current experiments to focus on core performance, DP can be seamlessly integrated. Specifically, before uploading, each client can apply the Gaussian mechanism to its update:

(20) $${\hat{W}}_{k}^{★(r)}=W_{k}^{★(r)}+N,\;\mathrm{where}\;N∼N\;\left(0,\;\sigma^{2}I\right).$$

The noise scale σ is calibrated based on the sensitivity $\Delta =\max_{k}{∥W_{k}^{★(r)}∥}_{F}$, the privacy budget ε, and the failure probability δ, according to $\sigma \geq \Delta \sqrt{2\ln(1.25/\delta )}/\varepsilon$. This ensures that any single client’s contribution is protected under a rigorous (ε,δ)-DP guarantee. A detailed empirical study of the accuracy-privacy trade-off is a valuable direction for future work.

### 3.3. Convergence and Complexity Analysis

In this section, we provide a theoretical analysis of the convergence properties and computational complexity of the proposed Fed-MUFSHT framework.

#### 3.3.1. Convergence Analysis

Providing a rigorous mathematical proof of convergence for a complex, hybrid framework such as Fed-MUFSHT is non-trivial. Instead, we offer a theoretical justification by analyzing the convergence properties of its constituent components: the inner-layer TL module, the outer-layer HC-THRO optimizer, and the global federated aggregation mechanism.

(1) Convergence of the inner-layer TL optimization. The convergence of the inner-layer optimization is well-established, as it adopts the alternating optimization strategy from the TIME-FS framework, whose convergence was proven in the original work. This strategy iteratively updates variables such as the low-dimensional representations $\left\{Z^{\left(v\right)}\right\}$ and the CP tensor factors (M,P,A) while keeping others fixed. For example, the updates for P and A are standard alternating least-squares subproblems, each admitting a closed-form solution. Since each step of the inner-layer update ensures that the objective function in Equation (14) is non-increasing, and the objective value is bounded below by zero, the sequence is guaranteed to converge to a stationary point.

(2) Convergence properties of the outer-layer HC-THRO. The outer-layer optimization is driven by HC-THRO, a metaheuristic algorithm. While formal convergence proofs for metaheuristics are often intractable, we highlight a crucial point: the convergence of the foundational THRO algorithm has been theoretically established by Wang et al. [28]. Our HC-THRO builds upon this proven foundation by integrating two key mechanisms to balance exploration and exploitation: HCL and ACM. The HCL mechanism (Equation (4)) promotes global exploration to avoid local optima, while the ACM mechanism (Equation (5)) enhances local exploitation to accelerate convergence toward high-quality solutions. This synergy is designed to progressively guide the search toward superior regions of the solution space. At the end of Section 4.2 of the empirical results, the results show stable convergence, which is consistent with the expected behavior of such a well-structured evolutionary algorithm.

(3) Convergence of the global federated aggregation. At the global level, Fed-MUFSHT employs a weighted model aggregation strategy on the server (Equation (17)), which is a variant of the widely-adopted Federated Averaging (FedAvg) algorithm [46]. The convergence of FedAvg and its variants has been extensively studied in the federated learning literature. Under standard assumptions, such as the smoothness of the loss function and bounded variance of client updates (i.e., a bounded degree of Non-IID data), FedAvg-based frameworks are proven to converge to a neighborhood of the global optimum [47]. In our framework, the local models (represented by $W_{k}^{★(r)}$) are optimized on local data for a set number of iterations before being aggregated by the server. This iterative process of local optimization and global aggregation adheres to the established convergence patterns of federated optimization.

(4) Overall framework convergence. Taken together, these components form a hierarchical optimization system where each layer exhibits strong convergence characteristics. The inner TL module is provably convergent to a stationary point. The outer layer employs HC-THRO, a robust metaheuristic built on a convergent foundation and designed for an effective global-local search. The global federated mechanism follows a provably convergent paradigm. The tight coupling of these components, where the output of one layer guides the next, fosters a stable optimization process. The empirical results in Figure 5, which show monotonic convergence of the objective function, empirically validate this theoretical justification.

#### 3.3.2. Computational Cost Evaluation

We examine the runtime demands of Fed-MUFSHT within the federated learning framework, featuring client-local training alongside central model fusion at the server. Let *K* denote total clients and $K_{b}\leq K$ the active subset. For each participant, data spans *V* views with $d_{v}$ features per view and *n* instances overall. The aggregate feature size is $D=\sum_{v}d_{v}$, while a common reduced dimension *r* is shared across views.

The local optimization procedure performed at each client follows a two-phase architecture. In the first phase, denoted as Stage-1 (HC-THRO), we parameterize the process with $N_{p}$ as the population count for the competing groups—specifically, Tianji’s horses (*A*) and the King’s horses (*B*)—and $T_{\mathrm{out}}$ as the outer iteration limit. The subsequent Stage-2 (TL) phase is governed by $T_{\mathrm{in}}$ inner iterations, with *k* and *r* representing the embedding dimensionality and CP rank, respectively. Within the HC-THRO phase, each iteration integrates Hierarchical Competitive Learning updates combined with Adaptive Cognitive Mapping adjustments for $N_{p}$ individuals. The HCL position update necessitates measuring directional gaps between paired individuals across the two populations, with a complexity of $O(N_{p}Dc)$, where $D=\sum_{v=1}^{V}d_{v}$ denotes the cumulative feature dimension and *c* the latent size. This cost is mirrored by the cognitive refinement process, which involves historical and population-mean updates, while fitness evaluation across all views adds a minor O(Dc) factor per individual. Consequently, an outer iteration for HC-THRO demands $O(N_{p}Dc)$, resulting in an aggregate Stage-1 overhead of $O(T_{\mathrm{out}}N_{p}Dc)$. Regarding the TL stage, the computational load is primarily driven by: (i) optimizing the local projection matrices ${\left\{W^{\left(v\right)},Z^{\left(v\right)}\right\}}_{v=1}^{V}$ and (ii) conducting a CP decomposition on the embedding tensor $Z\in R^{k\times n\times V}$. The optimization of $\left\{W^{\left(v\right)},Z^{\left(v\right)}\right\}$ incurs an $O(Vd_{v}nc)$ cost, which is reducible to O(ncD). Given that the rank-*r* alternating least squares update for CP decomposition requires O(rVkn), a single TL iteration complexity is O(ncD+rVkn). Thus, Stage-2 accumulates to $O(T_{\mathrm{in}}\left(ncD+rVkn\right))$. Integrating both components, the total per-client computational complexity for a single federated communication round is formulated as:

(21) $$O\left(T_{\mathrm{out}}N_{p}Dc+T_{\mathrm{in}}\left(ncD+rVkn\right)\right).$$

In each round, the server receives from every participating client a local optimal feature-weight matrix (of dimension D×c) and a scalar NMI score for secure aggregation. The server computes the normalized aggregation weights $\gamma_{k}=q_{k}/\sum_{j=1}^{K_{r}}q_{j}$ in $O(K_{r})$ time, and aggregates $K_{r}$ feature-weight matrices at a cost of $O(K_{r}Dc).$ Broadcasting the aggregated result to clients incurs a serialization cost of the same order.

Across *R* federated communication rounds, the total computational complexity equals the sum of client-side computation over all participating clients plus server-side aggregation, given by

(22) $$O\;\left(\sum_{r=1}^{R}K_{r}\left(T_{\mathrm{out}}N_{p}Dc+T_{\mathrm{in}}\left(ncD+rVkn\right)\right)\;+\;\sum_{r=1}^{R}K_{r}Dc\right).$$

When all *K* clients participate in every federated round (i.e., $K_{r}=K$), the complexity simplifies to

(23) $$O\;\left(RK\left(T_{\mathrm{out}}N_{p}Dc+T_{\mathrm{in}}\left(ncD+rVkn\right)\right)\;+\;RKDc\right),$$

where the last term represents the server-side aggregation and broadcasting operations.

#### 3.3.3. Communication Overhead

In each round, each participating client uploads a feature-weight matrix of size D×c and a scalar NMI value and receives the aggregated $\tilde{W}\in R^{D\times c}$. Thus, the communication cost per round is $O(K_{r}Dc)$ (uplink) plus $O(K_{r}Dc)$ (downlink), i.e., $O(K_{r}Dc)$ up to constant factors.

## 4. Experiments

This section conducts comprehensive experiments to evaluate the effectiveness and efficiency of the proposed Fed-MUFSHT framework. Specifically, the evaluation covers benchmark dataset descriptions, competing method introductions, hyperparameter configurations, ablation studies, convergence analysis, and statistical significance tests, all of which are designed to validate the superiority of Fed-MUFSHT over existing approaches.

### 4.1. Setup Details

In this section, we outline data sources, computing setup, baselines, tuning choices, and evaluation protocols.

#### 4.1.1. Details of Datasets

To examine the performance of the proposed Fed-MUFSHT in realistic application scenarios, we employ eight real-world multi-view datasets spanning image object recognition, document classification, handwritten digit analysis, and face recognition [10]. The principal characteristics of these datasets are summarized in Table 2.

#### 4.1.2. Discussion on Non-IID Scenarios

While a comprehensive empirical study on Non-IID data is planned for future work, we provide a theoretical justification for why the design of Fed-MUFSHT is inherently more robust to statistical heterogeneity compared to existing methods like Fed-IMUFS. This robustness stems from two key architectural advantages in our aggregation and distribution strategy:

(i) Advanced quality-aware weighting. A critical distinction lies in the weighting mechanism. While both methods use NMI scores, Fed-IMUFS employs simple linear normalization. In contrast, Fed-MUFSHT utilizes a more robust and flexible softmax function with a temperature parameter τ (Equation (16)). This provides more principled control over the influence of client updates. In scenarios with high statistical heterogeneity, where some local models may be of poor quality, a small τ can sharply increase the weight of high-performing clients while effectively silencing the detrimental ones. Conversely, a large τ can smooth the weights towards uniform averaging. This adaptability makes our aggregation strategy more resilient to the diverse model qualities arising from Non-IID data.
(ii) Personalized model distribution. Perhaps the most fundamental advantage is our personalized model distribution strategy (Equation (18)), a feature entirely absent in Fed-IMUFS. Instead of broadcasting a single, generic global model to all clients, we provide each client *k* with a personalized initialization $W_{k}^{0(r+1)}=\eta \;{\tilde{W}}^{\left(r\right)}+\left(1-\eta \right)\;W_{k}^{★(r)}$. This allows each client to retain a portion of its locally adapted knowledge while incorporating global consensus. This mechanism is crucial for mitigating “client drift” in Non-IID settings, as it prevents a generalized global model from completely overwriting specialized local knowledge, thereby preserving performance on heterogeneous local data.

Taken together, these two design choices—the adaptive softmax weighting and the personalized distribution—provide Fed-MUFSHT with a superior intrinsic capability to handle the challenges posed by Non-IID data.

#### 4.1.3. Hardware and Runtime Platform

MATLAB R2022a was used for all experimental analyses on a Windows desktop system. The computing device featured a 3.40 GHz Intel Core i7-13700KF CPU, 32 GB of DDR4 RAM, an NVIDIA RTX 3060 GPU equipped with 12 GB of memory, and a 2.29 TB solid-state disk. The available hardware was sufficient for supporting the optimization and learning procedures examined in this work.

#### 4.1.4. Comparative Baselines and Hyperparameters

To rigorously assess the proposed Fed-MUFSHT framework, we compare it with the only existing federated MUFS approach, Fed-IMUFS [43], as well as several representative centralized MUFS methods. Specifically, our centralized baselines include the state-of-the-art TIME-FS [23] and the widely used algorithms TRCA-CGL [8], JMVFG [18], CDMvFS [19], SDFS [20], and SCMvFS [48]. In addition, an “AllFea” reference method is introduced, where performance is assessed using the original feature space without dimensionality reduction.

Regarding the hyperparameter selection for Fed-MUFSHT, the federated communication rounds are capped at 5. Following the empirical recommendations in [23,43] and the sensitivity study reported in Section 4.4, the main parameters of Fed-MUFSHT are chosen as λ=0.1, τ=1, β=0.01, and μ=0.5. Furthermore, following Reference [43], we set the iteration threshold to 100 for the initial HC-THRO optimization stage and assign a population size of P=30. Finally, for all competing baselines, we strictly adhere to the optimal parameter configurations reported in their respective original literature.

#### 4.1.5. Comparison Protocol

A unified comparison setting is used throughout the experiments to minimize evaluation bias. For methods that cannot naturally accommodate incomplete multi-view samples, additional preprocessing is required before feature selection. In particular, except for TIME-FS and Fed-IMUFS, all baseline methods first replace missing entries with the corresponding mean values. The centralized methods are assessed in a favorable setting where information from all views is collected together. Under this condition, the complete sample set is available in a single repository, and missing elements are repaired beforehand whenever necessary. Because these methods operate with full access to global data, their results may be treated as a reference ceiling.

In contrast, Fed-MUFSHT is implemented under a distributed federated scheme. The full dataset is divided at random across *K* clients, and each client performs computation using only its own local data, with no direct exposure of raw features to the server or to other clients. Such a protocol better matches privacy-sensitive applications. After feature learning, all methods are evaluated through *k*-means clustering. Performance is assessed in terms of clustering accuracy (ACC) and NMI [23]. Each result corresponds to the average over 30 independent runs.

In addition, Fed-MUFSHT is further examined under a more realistic decentralized setting. In this case, the data is partitioned randomly among *K* clients, with each client’s original samples kept on-device and never exchanged, thereby satisfying privacy requirements. Under this setting, we perform an extensive sensitivity study over four main hyperparameters: the structure preservation coefficient λ∈{0.01,0.05,0.1,0.15,0.20,0.25}, the CP-based alignment weight τ∈{0.01,0.10,1,10,100}, the sparsity control factor β∈{0.001,0.005,0.01,0.05}, and the imputation penalty μ∈{0.1,0.3,0.5,0.7,0.9}.

### 4.2. Overall Performance Analysis of Fed-MUFSHT

To guarantee an unbiased assessment, a standardized preprocessing and evaluation pipeline is enforced across all comparative baselines. With the exception of TIME-FS, which handles raw inputs natively, all other methods operate on homogenized data representations. For centralized competitors, we adhere to a conventional benchmarking protocol wherein training occurs on a consolidated dataset formed by merging all samples and view-specific attributes. This setup presumes unrestricted access to global information, thereby serving as an idealized performance ceiling or upper-bound reference.

**Figure 3:**
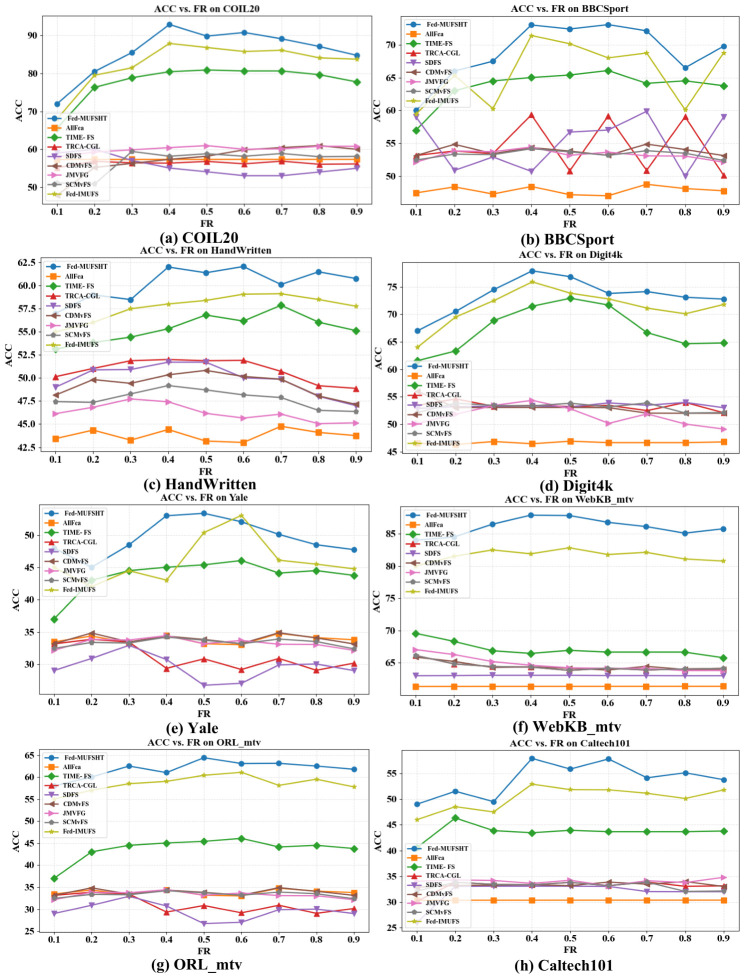
Comparison of ACC across eight datasets under varying feature selection ratios with 5 participating clients.

**Figure 4:**
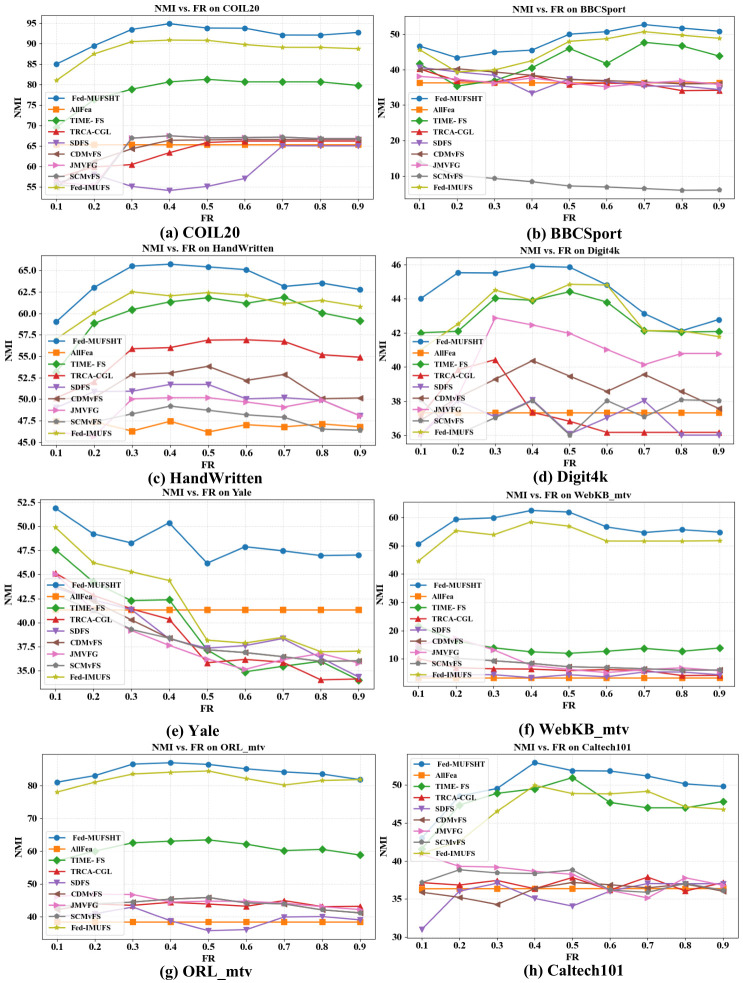
Comparison of NMI across eight datasets under varying feature selection ratios with 5 participating clients.

As the optimal number of selected features is generally unavailable in unsupervised settings [43], we vary the feature retention ratio (FR) from 10% to 90% at 10% intervals to evaluate the robustness of each method under different degrees of dimensionality reduction. All approaches are ultimately assessed via *k*-means clustering, with ACC and NMI [23] serving as the primary evaluation metrics. Figure 3 and Figure 4 illustrate the ACC and NMI outcomes across different FR, ranging from 10% to 90%. As evident from the results, Fed-MUFSHT consistently demonstrates superior performance compared to all competing approaches across nearly all FR levels. In particular, on WebKB_mtv, ORL_mtv, and Caltech101, the average gains of Fed-MUFSHT exceed 5% in both ACC and NMI when compared with the strongest federated competitor (e.g., Fed-IMUFS) and the best-performing centralized method (TIME-FS). On COIL20, Digit4k, and HandWritten, Fed-MUFSHT surpasses Fed-IMUFS, the second-best approach, by more than 3% on average. Similar advantages are also observed on the remaining datasets, where Fed-MUFSHT continues to achieve better clustering quality than the alternatives in terms of both ACC and NMI. These findings indicate that the proposed method is more effective at identifying discriminative and stable information from multi-view data, leading to more reliable clustering behavior in privacy-aware federated environments.

To examine computational cost and runtime efficiency of Fed-MUFSHT, empirical measurements were performed across three benchmark datasets—namely BBCSport, WebKB, and Digit4k—under a five-client federated architecture. Table 3 reports the average running time of Fed-MUFSTH and all baselines, as well as their theoretical time complexities. According to the results, Fed-MUFSTH exhibits linear time complexity with respect to the per-view sample size *n*.

On the BBCSport and WebKB benchmarks, our method exhibits marginally reduced execution time compared to TIME-FS, whereas on Digit4k, it attains the minimal runtime and superior overall efficiency among all competitors.

**Figure 5:**
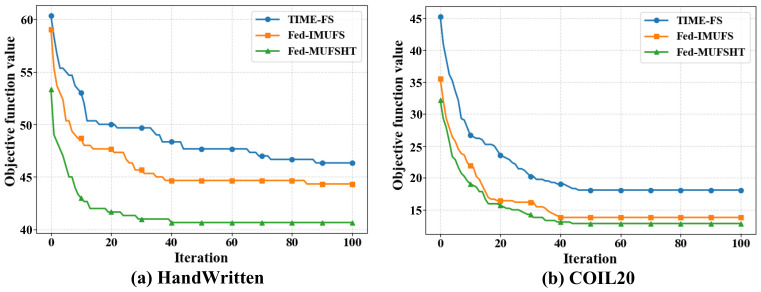
Convergence curves of Fed-MUFSHT and two competitive leading approaches for HandWritten and COIL20.

Additionally, we employ the HandWritten and COIL20 datasets and consider two recent state-of-the-art approaches, Fed-IMUFS and TIME-FS, as baselines to evaluate the convergence performance of Fed-MUFSHT. As shown in Figure 5, Fed-MUFSHT achieves better optimal values and converges faster than these competing methods.

### 4.3. Ablation Study

To examine how different components affect the final performance of Fed-MUFSHT, we design a set of ablation experiments by comparing the complete framework with several reduced variants. Specifically, Proposed-I excludes the Stage-1 HC-THRO procedure and preserves only the Stage-2 TL module. Proposed-II removes the TL learning stage and performs optimization using only HC-THRO. Proposed-III substitutes HC-THRO in the first stage with the original THRO optimizer while keeping the second-stage TL process unchanged, so as to evaluate the role of the improvements introduced into HC-THRO. Proposed-IV discards the Hierarchical Competitive Learning component from HC-THRO, whereas Proposed-V eliminates the Adaptive Cognitive Mapping mechanism. Proposed-VI further replaces the original secure aggregation and distribution strategy, which relies on NMI and feature weighting, with a standard weighted averaging rule.

Table 4 summarizes the average outcomes from 30 runs on eight datasets under a FR of 40% and a five-client setting. The comparison demonstrates that every reduced version, from Proposed-I to Proposed-VI, performs noticeably below the complete Fed-MUFSHT framework. This observation suggests that each module contributes positively to the overall system, and that combining them is important for achieving strong performance.

### 4.4. Hyperparameter Sensitivity Analysis

**Figure 6:**
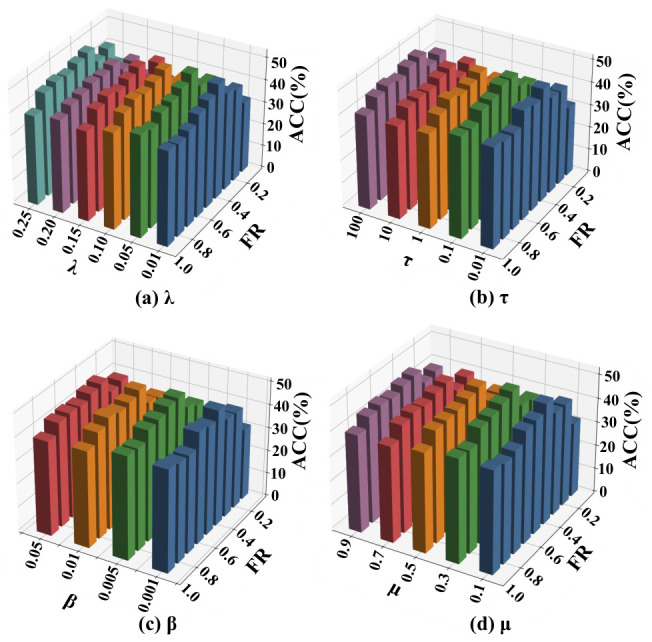
ACC of Fed-MUFSHT versus four key hyperparameters and FR on Yale.

**Figure 7:**
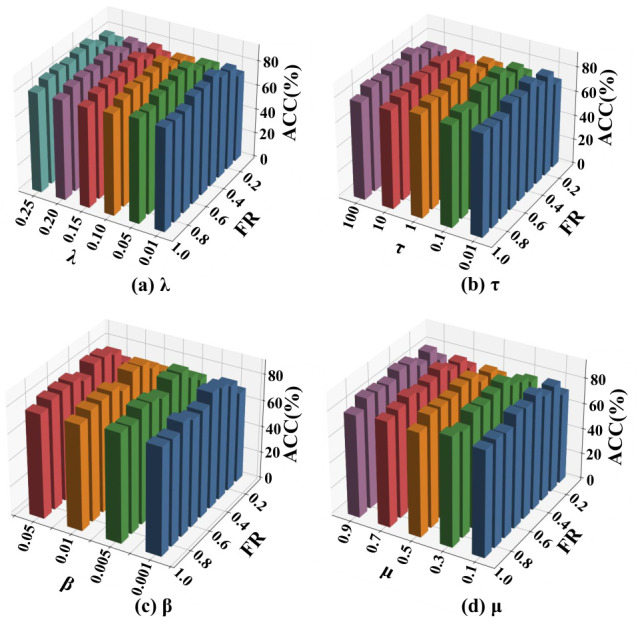
ACC of Fed-MUFSHT versus four key hyperparameters and FR on COIL20.

**Figure 8:**
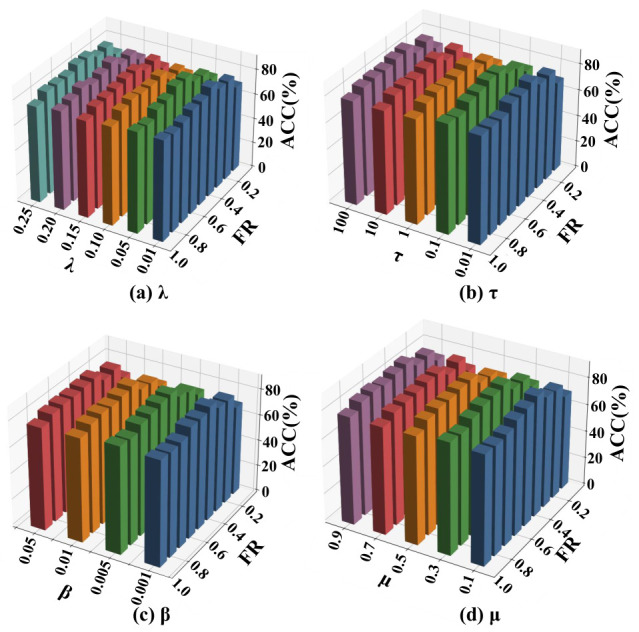
ACC of Fed-MUFSHT versus four key hyperparameters and FR on WebKB.

To evaluate the impact of key hyperparameters on the performance of Fed-MUFSHT, we conduct a sensitivity analysis on four core parameters: λ,τ,β, and μ. The experimental setup involves five participating clients, with the sample MR fixed at 40% and the FR varying from 10% to 90%. ACC is employed as the primary evaluation metric. Subplots (a)–(d) correspond to the sensitivity results of hyperparameters λ, τ, β, and μ, respectively. In each subplot, bars with different colors represent different values of the corresponding hyperparameter, where distinct colors are used to distinguish different candidate values for clear visualization. The horizontal axis represents the FR, and the vertical axis represents the ACC. Furthermore, we assess the performance of the model across three representative datasets with varying sample sizes—Yale, COIL20, and WebKB—under different hyperparameter configurations. Each reported result is obtained by averaging the outcomes of 30 independent runs, as illustrated in Figure 6, Figure 7 and Figure 8.

The experimental results demonstrate that variations in λ,τ,β, and μ exert only a minor influence on overall performance, indicating that Fed-MUFSHT exhibits strong robustness to parameter fluctuations. Notably, the model achieves optimal or near-optimal performance consistently across all three datasets when the hyperparameters are set to λ=0.1,τ=1,β=0.01, and μ=0.5. Consequently, these values are adopted as the default configuration for the subsequent experiments.

### 4.5. Statistical Significance Analysis

To verify that the superiority of Fed-MUFSHT is statistically reliable, we perform a non-parametric analysis under a controlled setting (FR = 40%, clients = 5, metric = ACC over eight datasets). We first conduct a Friedman test [43,49,50] to examine the overall difference among all eight methods, yielding $\chi_{F}^{2}=31.42$. With the Iman–Davenport correction for small *N*, this corresponds to $F_{F}=8.95$ ($p=1.2\times 10^{-6}<0.05$), rejecting the null hypothesis of equivalent performance.

Pairwise comparisons are then performed using the Wilcoxon signed-rank test with Holm–Bonferroni correction (α=0.05). Table 5 reports the win/tie/loss counts, Holm-adjusted *p*-values, and the matched-pairs effect size given by the rank-biserial correlation $r_{\mathrm{rb}}=\left(W^{+}-W^{-}\right)/\left(W^{+}+W^{-}\right)$. Following Cohen’s convention, |r|≤0.1 negligible, 0.1<|r|≤0.3 small, 0.3<|r|≤0.5 medium, and |r|>0.5 large. Symbols ‘+’/‘∼’/‘−’ indicate if Fed-MUFSHT is significantly better/comparable/worse than the baseline. Note: with only N=8 datasets, *p*-values are based on the asymptotic normal approximation; the smallest achievable exact two-tailed *p* is 0.0078.

Fed-MUFSHT significantly outperforms TIME-FS on six datasets, ties on one, and loses on one (Holm-adjusted $p=1.1413\times 10^{-2}$, $r_{\mathrm{rb}}=0.47$, medium effect). Against TRCA-CGL, SDFS, CDMvFS, JMVFG, and SCMvFS, it wins on all eight datasets with Holm-adjusted $p\in [4.1812\times 10^{-4},2.7432\times 10^{-2}]$ and large effects ($r_{\mathrm{rb}}\in \left[0.61,0.72\right]$). Compared with Fed-IMUFS, it wins on seven datasets and ties on one ($p=3.15\times 10^{-2}$, $r_{\mathrm{rb}}=0.54$, large effect).

## 5. Conclusions

This paper proposes the Fed-MUFSHT to tackle privacy preservation in distributed MUFS, alongside the longstanding challenges of suboptimal solution quality and sluggish convergence in conventional MUFS approaches. By innovatively integrating the HC-THRO for global feature exploration with TL for latent representation refinement, coupled with a Secure multi-party aggregation and distribution strategy based on NMI and feature weights, our method enables efficient collaborative training without the exchange of raw data. Extensive experimental results demonstrate that Fed-MUFSHT significantly outperforms state-of-the-art centralized and federated baselines in terms of FS solution quality, convergence stability, and privacy protection, thereby confirming its effectiveness and robustness for privacy-sensitive and complex multi-view data tasks.

Despite the promising results, two limitations should be acknowledged. First, the NMI-based weighting mechanism assumes that mutual information reliably reflects feature relevance; however, when data distributions are highly skewed or views contain predominantly noisy features, NMI estimates may become unreliable, potentially leading to suboptimal aggregation. Second, the current framework assumes homogeneous view structures across clients, limiting its applicability to heterogeneous scenarios with varying view counts or feature dimensions.

Future work will focus on developing adaptive weighting mechanisms robust to skewed or noisy distributions, conducting comprehensive empirical evaluations on Non-IID data, extending the framework to handle more intricate heterogeneous scenarios—such as varying view counts and disparate feature dimensions—and establishing a customized federated deep learning baseline for MUFS.

## Figures and Tables

**Table 1:** Structuralcomparison of Fed-MUFSHT with the most related works.

Aspect	TIME-FS [23]	Fed-IMUFS [43]	Fed-MUFSHT (Ours)
Learning paradigm	Centralized	Federated	Federated
Optimization strategy	Single-stage alternating optimization (ALS)	Two-stage: WOA + Tensor alternating learning	Two-stage synergistic: HC-THRO (outer) + TL (inner) with closed-loop feedback
Global search mechanism	None (prone to local optima)	Sparsity-guided WOA	HC-THRO with HCL & ACM
Exploration–exploitation balance	Relies solely on ALS initialization	Sparsity-guided WOA operators	Dual-mechanism: HCL for inter-population competition + ACM for cognitive refinement
Federated aggregation	Not applicable	Standard weighted averaging	NMI-based quality-aware softmax weighting with personalized redistribution
Inner–outer coupling	Not applicable	Sequential (loosely coupled)	Tightly coupled: TL output refines HC-THRO fitness; HC-THRO solution initializes TL

**Table 2:** Descriptionof datasets.

Datasets	Samples	Classes	Views	Features
COIL20	1440	20	3	30/19/30
BBCSport	544	5	2	7073/6935
HandWritten	544	5	2	4657/1125
Digit4k	2000	10	4	240/216/47/64
Yale	165	15	2	1024/3304/6750
WebKB	2100	21	3	540/640/256
ORL_mtv	400	40	3	4096/3304/6750
Caltech101	1474	7	6	48/40/254/1984/512/928

**Table 3:** Comparison of average execution duration (in s) and theoretical time complexity across three benchmark datasets. The most efficient results are emphasized in boldface. Here, $d=\sum_{v=1}^{V}d_{v}$ denotes the total feature dimensionality.

Methods	BBCSport	WebKB	Digit4k	Computational Cost
Fed-MUFSTH	7.30	35.23	15.40	$O\;\left(RK\left(T_{\mathrm{out}}N_{p}Dc+T_{\mathrm{in}}\left(ncD+rVkn\right)\right)\;+\;RKDc\right)$
TIME-FS	7.90	16.55	14.14	$O(dnc+c^{2}nV)$
TRCA-CGL	16.91	129.37	38.69	$O(n^{2}V^{2}+2n^{2}V\log\left(n\right)+d^{3})$
SDFS	29.65	197.19	53.45	$O\;\left(\sum_{v=1}^{V}\left(n^{3}+n^{2}d_{v}+nd_{v}^{2}+d_{v}^{2}\right)\right)$
CDMvFS	52.03	529.98	76.38	$O(Vn^{3}+\sum_{v=1}^{V}d_{v}^{2})$
JMVFG	9.22	73.78	56.45	$O\;\left(\sum_{v=1}^{V}d_{v}^{3}+n\sum_{v=1}^{V}d_{v}^{2}+n^{2}d\right)$
SCMvFS	23.05	258.15	65.64	$O(Vn^{3}+\sum_{v=1}^{V}d_{v}^{2})$
Fed-IMUFS	8.10	47.01	16.40	$O\;\left(RK[T_{1}Pdc+T_{2}\left(Vdnc+r\left(c+n+V\right)\right)]\right)$

**Table 4:** ACC (%) and NMI (%) comparison between Fed-MUFSHT and its ablation variants; * marks statistically significant gains based on the Wilcoxon test (p<0.05).

Datasets	Proposed		Proposed-I		Proposed-II		Proposed-III		Proposed-IV		Proposed-V		Proposed-VI	
	ACC	NMI	ACC	NMI	ACC	NMI	ACC	NMI	ACC	NMI	ACC	NMI	ACC	NMI
COIL20	91.57 *	97.15 *	86.82	92.98	85.97	91.14	87.43	93.12	88.06	92.84	86.64	91.73	88.41	93.03
BBCSport	74.55 *	47.12 *	70.81	44.97	71.98	45.14	71.45	44.11	71.05	45.85	70.63	43.72	71.42	44.23
HandWritten	78.18 *	46.43 *	57.95	65.23	56.62	59.77	59.84	62.09	60.21	61.84	59.45	60.37	61.72	62.35
Digit4k	77.65 *	46.64 *	72.39	41.14	71.81	40.08	73.51	43.12	72.96	42.74	71.26	41.33	73.19	42.53
Yale	53.65 *	50.64 *	50.39	47.14	49.80	47.07	51.51	48.11	51.96	46.78	50.27	47.38	50.18	46.52
WebKB	87.17 *	61.89 *	82.46	55.59	82.81	54.15	81.27	58.43	82.15	55.82	81.27	52.67	83.66	56.82
ORL_mtv	60.73 *	85.28 *	54.82	80.87	53.07	81.34	54.47	80.11	55.62	81.93	56.54	82.11	53.07	79.05
Caltech101	57.85 *	58.42 *	50.40	51.47	52.21	53.84	52.13	52.24	53.86	54.92	53.94	52.31	51.76	50.88

**Table 5:** Statistical comparison based on asymptotic Wilcoxon signed-rank test with Holm correction (α=0.05) over eight datasets. Effect size: rank-biserial $r_{\mathrm{rb}}$.

Baselines	Better	Similar	Worse	*p*-Value	Significance	Effect Size
Proposed—TIME-FS	6	1	1	1.1413 × 10^−2^	+	0.47
Proposed—TRCA-CGL	8	0	0	2.7432 × 10^−2^	+	0.71
Proposed—SDFS	8	0	0	4.3526 × 10^−3^	+	0.71
Proposed—CDMvFS	8	0	0	4.1812 × 10^−4^	+	0.72
Proposed—JMVFG	8	0	0	1.7351 × 10^−3^	+	0.61
Proposed—SCMvFS	8	0	0	4.2739 × 10^−3^	+	0.61
Proposed—Fed-IMUFS	7	1	0	3.1463 × 10^−2^	+	0.54

## Data Availability

Information required to reproduce the reported results is included in this manuscript, covering the main experimental setup and parameter specifications. No additional supporting materials are available.
